# Supplementary material for: The Prognostic and Predictive Value of SOX2+ Cell Densities in Patients Treated for Colorectal Cancer
Source: Cancers (Basel). 2020 Apr 29;12(5):1110. doi: 10.3390/cancers12051110 (PMC7280991; doi:10.3390/cancers12051110)
Supplement: Supplementary file 1 [file cancers-12-01110-s001.pdf]

Table S1. Correlations between clinicopathological variables and high SOX2 expression.

Variable	Stage II n = 445				Stage III n = 352			
	**SOX2^{High}, n(%)**	**OR**	**95%CI**	***p***	**SOX2^{High}, n(%)**	**OR**	**95%CI**	***p***
Age[1]		<0.01		0.802		0.01		0.095
T Stage								
T1	-				0 (0.0)	-	-	0.971
T2	-				1 (9.1)	0.80	0.04-14.9	0.881
T3	33 (7.9)	0.66	0.19-2.30	0.509	42 (12.7)	1.16	0.14-9.53	0.888
T4	3 (11.5)	1.00			1 (11.1)	1.00		
Localization								
Not Reported	0 (0.0)	-	-	0.983	-			
Proximal	20 (10.0)	1.70	0.69-4.14	0.249	22 (15.7)	1.40	0.66-2.97	0.385
Distal	9 (7.7)	1.26	0.45-3.51	0.656	10 (9.1)	0.75	0.31-1.82	0.525
Rectal	7 (6.2)	1.00			12 (11.8)	1.00		
Vascular Invasion								
Not Reported	2 (13.3)	1.76	0.38-8.19	0.470	-			
Present	5 (7.3)	0.89	0.38-8.19	0.824	20 (12.5)	1.00	0.53-1.89	1.00
Absent	39 (8.0)	1.00			24 (12.5)	1.00		
PNI								
Not Reported	2 (10.0)	1.26	0.28-5.67	0.763	-			
Present	1 (5.6)	0.67	0.09-5.17	0.698	5 (12.8)	1.03	0.38-2.80	0.948
Absent	33 (8.1)	1.00			39 (12.5)	1.00		
Grade								
Not Reported	0 (0.0)	-	-	0.990	0 (0.0)	-	-	0.992
High	6 (13.3)	1.87	0.73-4.78	0.190	6 (16.7)	1.45	0.57-3.72	0.436
Low	30 (7.6)	1.00			38 (12.1)	1.00		
MMR								
Not Reported	0 (0.0)	-	-	0.988	2 (25.0)	2.36	0.46-12.1	0.303
Deficient	6 (9.5)	1.21	0.48-3.03	0.689	2 (9.5)	0.75	0.17-3.32	0.699
Proficient	30 (8.0)	1.00			40 (12.4)	1.00		

[1]Continuous variable analyzed with linear regression. R^2 value reported instead of OR and p-values calculated using a t-test.

PNI Perineural Invasion; MMR Mismatch repair status

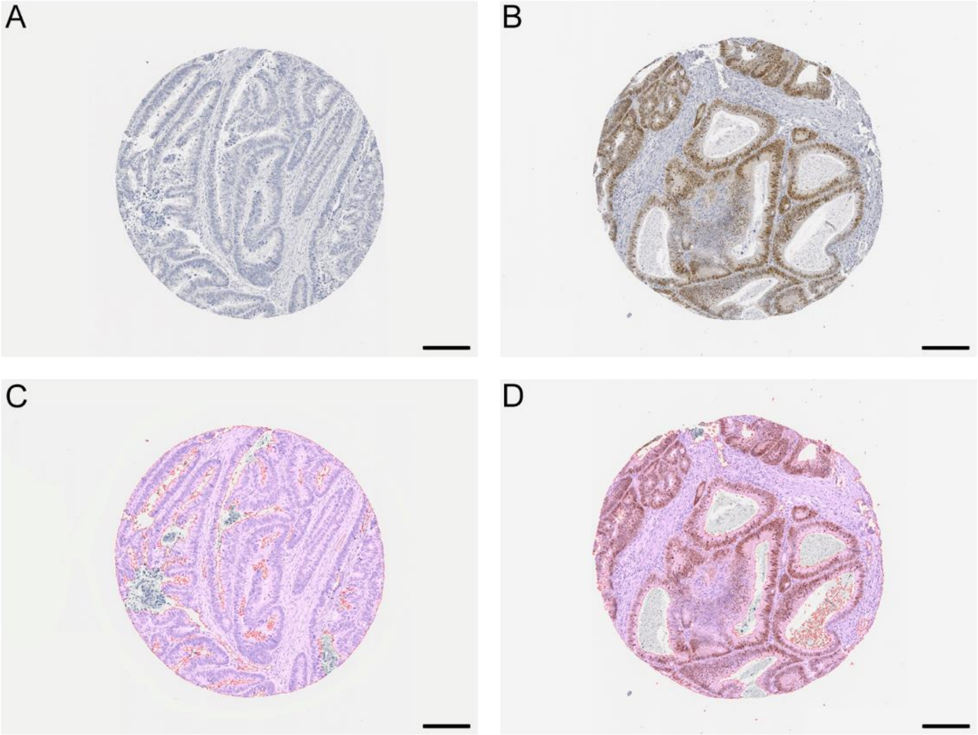

Figure S1: Tissue detection in StrataQuest software via creation of a digital mask. A digital 'mask' is created to measure the area of tissue for quantification of cell densities. The tissue mask is automatically generated by StrataQuest software via conversion of the scanned image from RGB to grayscale and then application of an intensity threshold. Manual adjustments are made to the tissue mask to remove necrotic areas and/or staining artefacts. Only nuclei present within the tissue mask are quantified. Representative tumor cores with low (A) and high (B) SOX2 densities, respectively, with corresponding overlaid tissue masks are shown (C-D; purple color). Tissue mask generation is based on haematoxylin staining so is not affected by the level of DAB staining. Scale bars 200μm.

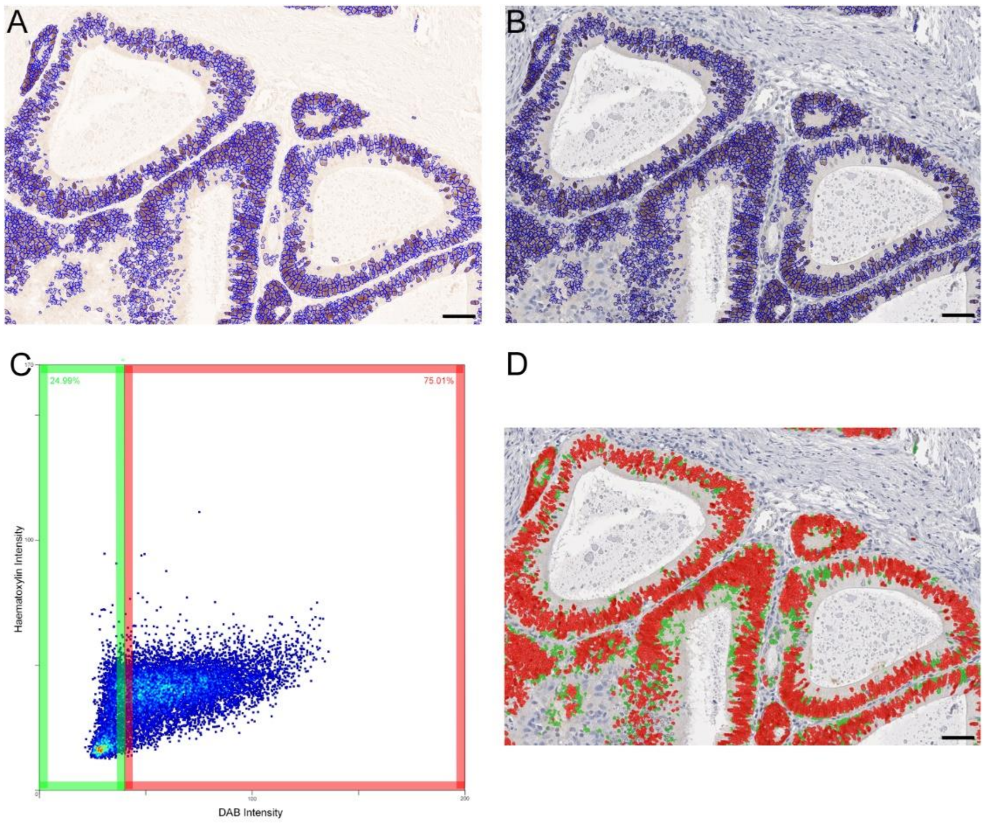

Figure S2: StrataQuest workflow for detection of SOX2+ nuclei and thresholding. Inbuilt color deconvolution algorithms within StrataQuest software separate SOX2 DAB (brown) staining from haematoxylin (blue) staining to produce grayscale images for each channel. Nuclear segmentation was then performed on the resulting grayscale DAB image to detect brown-stained nuclei. This is possible as SOX2 expression is localized to the nucleus. Segmented nuclear masks overlaid onto the DAB (brown) channel (A) and the original color image (B) allow visualization of the segmentation algorithm. The software then calculates parameters such as nuclear size, haematoxylin intensity and DAB intensity for each nuclear mask, which are reported on a scattergram, with each dot representing a single nuclear mask. A scattergram displaying DAB intensity vs haematoxylin intensity is used to threshold and accurately detect SOX2+ stained nuclei (C). Gated nuclei from the scattergram (C) can be visualized on an image of segmented nuclear masks overlaid onto the RGB image (D). Red nuclei represent SOX2+ cells (red gate on C) and green nuclei are SOX2- cells (green gate on C). Scale bars 50μm.
